# Feasibility of In-home Saliva Collection of Cortisol and DHEA-S as a Biomarker of Stress in Dementia Care Dyads

**DOI:** 10.1093/geroni/igab046.3605

**Published:** 2021-12-17

**Authors:** Azita Emami, Gabriella Engström, Hyejin Kim

**Affiliations:** 1 University of Washington School of Nursing, University of Washington, Washington, United States; 2 Dalarna University, Falun, Dalarnas Lan, Sweden; 3 University of Washington School of Nursing, Seattle, Washington, United States

## Abstract

Dementia afflicts affected individuals and their family caregivers worldwide. Although a non-pharmacological intervention has been recommended as a first-line approach to minimize adverse outcomes (e.g., stress) in dementia care dyads (persons with dementia [PWD] and their family caregivers), most evaluations of such interventions have relied on subjective (e.g., self- or proxy-report) rather than objective (e.g., biomarkers) measures. We aimed to explore the feasibility of saliva collection of cortisol and dehydroepiandrosterone sulfate (DHEA-S) as a non-intrusive method in dementia care dyads. Dementia care dyads living at home were recruited from the memory center in Sweden. Prior to the saliva collection, participants received a one-hour education session with a hands-on demonstration led by a trained study coordinator. Participants were instructed to collect saliva three times (two for morning, one for evening)/day, five days/week for eight consecutive weeks. Out of 32 care dyads (32 PWD and 32 family caregivers), 24 (75.0%) completed the saliva collection. On average, 105.5 (87.92%) and 105.9 (88.25%) samples were collected from PWD and family caregivers during eight weeks. There were no statistically significant differences (p>0.05) in the average number of saliva samples (i.e., total samples, morning or evening samples) between PWD and family caregivers. The findings of this pilot study showed that saliva collection of cortisol and DHEA-S as a stress measurement was feasible in dementia care dyads living at home. Robust and person-centered procedures, tailored educational materials, and effective communication with dementia care dyads should be considered in future biomarker research on stress in dementia care dyads.

